# Time to first optimal glycemic control and its predictors among type 1 diabetic children in Bahir Dar city public referral hospitals, North West Ethiopia: a retrospective follow up study

**DOI:** 10.1186/s12887-022-03604-8

**Published:** 2022-09-24

**Authors:** Fentahun Meseret, Amare Belachew, Getasew Tesfa, Teshale Mengesha, Tsegasew Embiale, Ayichew Alemu, Melsew Dagne

**Affiliations:** 1 Department of Pediatrics and Child Health Nursing, Haramaya University, College of Health and Medical Science, School of Nursing, and Midwifery, P.O. Box 235, Harar, Ethiopia; 2 Bahir Dar University, College of Medicine and Health Sciences, Department of Pediatrics and Child Health Nursing, P.O. Box 79, Bahir Dar, Ethiopia; 3 Department of Pediatrics and Child Health Nursing, Dire Dawa University, Deri Dewa, Ethiopia; 4 Department of Adult Health Nursing, Woldia University, College of Health and Medical Science, Woldiya, Ethiopia

**Keywords:** Type 1 diabetes mellitus, First optimal glycemic control, Time, Children, Ethiopia

## Abstract

**Background:**

Recognizing the level of glycemic control of a client is an important measure/tool to prevent acquiring complications and risk of death from diabetes. However, the other most important variable, which is the time that the patient stayed in that poor glycemic level before reaching optimal glycemic control, has not been studied so far. Therefore, this study aim to estimate time to first optimal glycemic control and identify predictors among type 1 diabetic children in Bahir Dar city public referral hospitals, Northwest, Ethiopia, 2021.

**Methods:**

A Retrospective cohort study was conducted at Bahir Dar city public referral hospitals among a randomly selected sample of 385 patients with type 1 diabetes who were on follow up from January 1, 2016 to February30, 2021.Data were collected by using a data abstraction tool and then entered into Epi-data version 4.6 and exported into STATA 14.0 statistical software. Descriptive statistics, Kaplan Meier plots and median survival times, Log-rank test and Cox-proportional hazard regression were used for reporting the findings of this study. After performing Cox-proportional hazard regression, model goodness-of-fit and assumptions were checked. Finally, the association between independent variables and time to first optimal glycemic control in months was assessed using the multivariable Cox Proportional Hazard model and variables with a *p*-value < 0.05 were considered as statistically significant.

**Results:**

Median survival time to first optimal glycemic control among type 1 diabetic clients was 8 months (95%CI: 6.9–8.9). The first optimal glycemic achievement rate was 8.2 (95%CI: 7.2–9.2) per 100 person/month observation. Factors that affect time to first optimal glycemic control were age > 10–14 years (AHR = 0.32;95%CI = 0.19–0.55), increased weight (AHR = 0.96;95%CI = 0.94–0.99), having primary care giver (AHR = 2.09;95%CI = 1.39–3.13), insulin dose (AHR = 1.05;95%CI = 1.03–1.08), duration of diabetes ≥4 years (AHR = 0.64;95%CI = 0.44–0.94), adherence to diabetic care (AHR = 9.72;95%CI = 6.09–15.51), carbohydrate counting (AHR = 2.43;95%CI = 1.12–5.26), and comorbidity (AHR = 0.72;95%CI = 0.53–0.98).

**Conclusion:**

The median survival time to first optimal glycemic control in this study was long. Age, weight, primary care giver, insulin dose, duration of diabetes, adherence, and carbohydrate counting, including history of comorbidity were determinant factors. Giving attention for overweight and comorbid illness prevention, increasing either the dose or frequency of insulin during initial treatment; counseling parent (for both the mother and father) about adherence to diabetic care focusing on insulin drugs and how to audit their children’s diet as prescription helps to reduce the length of glycemic control.

## Background

Glycemic control (GC) is a level of glucose in diabetic clients [1]; Diabetes mellitus (DM) pandemic has become one of the largest global health emergencies among non-communicable diseases in this century [2]. In many countries, over 500,000 children < 15 years old are diagnosed with type one diabetes mellitus (T1DM) [3–5] with an average incidence of 3 to 4% per year worldwide [6, 7]. This increment is also noted more alarmingly in developing countries [6–9].

Although there are lots of advanced management of T1DM, more than 70% of them were unable to maintain their glycaemia [7, 9]. More over noncompliance rate escalating 50% highlights the need for focusing on timely optimal glycemic control (OGC) [7]. Many children had also suffered from T1DM which is associated with high morbidity, mortality rate, and most of the time the poor have been highly affected by this disease [6, 10, 11]. Both in developed and developing nations, the prognosis for children with T1DM is poor [9]. As a result, OGC oscillated from 2.6–9.8% in Tanzania [12, 13], 24% in Cameron [9], 24% in Sudan [14] 28% in Egypt [15]; 20.9% in Jordan [16] 39.1% in Saudi Arabia [17] and 33,36% in California [18] and Bulgaria [19] respectively. Many are not detected and those diagnosed have dramatically reduced their life expectancy by 1 year, [20]. GC was challenging among type one patients (82.9%) as compared with type two diabetics (57.7%) [9].

A variety of factors that predict glucose control in children with T1DM have been documented [21]. High proportion of patients with uncontrolled glycemic level in developed nations were due to sociodemographic factors(pubertal age, female sex, absence of direct mother care), concomitant disease (Coexistence of type 1 diabetes and other autoimmune diseases), dietary noncompliance and other psychological factors [11, 16, 17, 20, 22, 23]. Whereas, health care system with limited resources, lack of trained health personnel/diabetic care team, having a mother as the primary caregiver, less than 3 clinic visits per year,), in subjects with lipohypertrophy, known celiac disease, age at diabetes onset and diabetes duration, insulin treatment regimen, adherence to insulin therapy, practice of self-monitoring, in ability of the patient or family to use and afford treatment expenditures were identified risk factors in developing countries [6, 7, 9, 10, 24].

Uncontrolled glycemic situation results in complications which can hurt many parts of the body, including growth failure later in time [3, 24]. As a result, both acute and chronic complications were reported in different studies [24]. Adverse effects like lipodystrophy are one of the clinical complications which may occur related to insulin injection and lead to insulin absorption problems, which ultimately can hinder first OGC [12]. The most common complications prior to 3 months were hypoglycemia (21–42%) followed by 31.5–39% of diabetic ketoacidosis (DKA), 10.5–32.9% of nephropathy, 13.6%of neuropathy, 10.5% of convulsion, 10.3% of retinopathy [25, 26]. Sustained abnormal blood sugar fluctuation for periods of greater than 2 months can also contribute to high burden of the disease, hospitalization, and negative consequences of disease outcomes [13].

Similarly, a study in Ethiopia highlights the difficulty of achieving GC early in time. As a result, early occurrences of both retinopathy and maculopathy among diabetic children were reported [8]. Another study in Ethiopia specifically in Gojjam, also indicates 58.5% DKA among 354 T1DM children with the incidence rate of 2.27/100 children/month of observation [27].

However, strict glycemic control minimizes the incidence and progression of such possible complication [9, 20]. The Diabetes Control and Complication Trial (DCCT) and the follow-up study Epidemiology of Diabetes Interventions and Complications (EDIC) show that, good GC with short duration delays the development of both acute and chronic complications in T1DM patients by 35–76% [6]. Novel treatment are emerging to manage T1DM with the ultimate goal being to achieve GC, limit weight gain, reduce comorbidities and improve quality of life [4]. T1DM treatment is based on frequent monitoring of blood glucose and administration of insulin, in line with their meal and exercise [13, 28, 29]. It was recommended that T1DM children should check their blood glucose at least four times a day [3] either by their primary care giver or by their own based upon their development level. And which is expected to bring 26.2% satisfactory GC level [4, 29]. People with diabetes can live longer and have a healthy life if their diabetes is become aware of early and well-managed by a multidisciplinary approach with the allocation of accessible resources [7]. Being updated about the recent diabetes care can also help in improving first GC [30].

In Ethiopia, a little studies were conducted to recognize levels of GC among type one diabetic children [11]. However, the other most important parameter, which is the time, in which, the patient stayed on that poor glycemic level before reaching OGC, has not been studied so far. If efforts are not made to recognize the contributing factors for OGC within a possible time frame, the number of children affected will continue to grow and this in turn leads to an emotional and economic burden on both the clients and the families at large [3]. And it will also disturb the sustainability of our health care system, which is still over-burdened with communicable diseases.

Therefore, this study was aimed to estimate the time to first OGC among type 1 diabetic children in Bahir Dar city public referral hospitals, Northwest, Ethiopia.

## Methods and materials

### Study area and period

The study was conducted in Bahir Dar city; located 565Km far from Addis Ababa, the capital city of Ethiopia, in Amhara national regional state, North West Ethiopia. In Bahir Dar city there are two public referral hospitals, one primary hospitals, ten health center, and four private hospitals. This study was conducted in the two public referral hospitals, namely: Felege Hiwot Comprehensive Specialized Referral Hospital (FHCSH) and Tibebe Ghion Specialized Teaching Hospital (TGSTH). Each of these hospitals can be expected to serve more than 10 million populations in their catchment area. The study period address from1stJanuary, 2016 to February 30 /2021. We have planned to conduct this study in this period of interval to be in line with World Health Organization (WHO) global report on diabetes in 2016.The WHO diabetes report since 2016 identified a huge gap in diagnosis, management approach, and data management related to pediatric DM, particularly in developing countries, with possible recommendations to narrow such observed challenges since that time [1].

### Study design

An institution based retrospective follow-up study was employed.

### Source population

The source population were all type 1 diabetes mellitus, children < 15 years old who had follow-up (on antidiabetic treatment) at the diabetes clinic of the two referral hospitals.

### Study population

The study population were all type 1 diabetes mellitus children < 15 years old who were on follow-up (on antidiabetic treatment) during the study period.

### Study unit

All type one diabetic children’s charts that were selected randomly for investigation.

### Inclusion criteria

Children age less than 15 years old and diagnosed with T1DM with regular follow-up and had at least one HbA1c and/or a 3 month consecutive measurements of fasting blood sugar (FBS) with clear date of diagnosis between January 1/2016 to February 30/2021 were included.

### Exclusion criteria

Children’s medical record/chart with incomplete information (such as HbA1c/average FBS and other relevant predictors like age with date of diagnosis, sex, treatment modality, frequency of follow up visit and last visit health condition of the children), those having less than 3 month follow up during the study period and those cases transferred in with unclear date of diagnosis from other institution were excluded from the study.

### Sample size determination

Sample size was determined by double proportion formula after taking of predictors associated to optimal glycemic control from previous study conducted by retrospective cohort design [16] with the help of epi info version 7 by considering the following statistical assumptions: 95% Confidence Interval (CI), power 80%, percent of outcome in unexposed group 8.93%,risk ratio 0.253; marginal error 5% [16]. The calculated total sample size is 378;then by adding 10% for missed client chart, the final sample size becomes 416.

### Sampling technique and procedure

The study participants were selected from the registration book. The medical records of children who were on follow-up with type one diabetes mellitus from January 2016 to February 2021 were selected. A total of 721 children were recorded from the registration books of the two referral hospitals. Of which 416 cards were sampled using a simple random sampling technique by a computer generated method. Finally, cards that fulfilled the criteria were reviewed.

### Dependent variables

Time to first optimal glycemic control.

### Independent variables

Socio-demographic (age, gender, Residence); Institutional related variable (frequency of clinic visit); Diabetic related variables (duration of diabetes, diabetes-related complication); Comorbidities (preceding infections and other pathology) and treatment-related variables (insulin therapy and adherence, noncompliance and other self-monitoring practice).

Age of the participants, frequency of glycemic control, body mass index and duration of diabetes were categorized into groups in order to align with the other literatures [16].

### Operational definitions

#### Optimal glycemic control

OGC is defined as the three consecutive month HbA1c < 7.5% with more or less stringent glycemic goals for individual clients based on age/life expectancy, comorbid condition, advanced complication, hypoglycemia unawareness and individual patient considerations [3, 5]. GC followed by the diagnosis was reflected by optimal and poor metabolic control as mean HbA1c < 7.5 and > 7.5% respectively and /or average FBS level between 80 and 150 mg/dl and either < 80 or > 150 mg/dl respectively [3, 5, 31]; HbA1c can be calculated from the following formula, if HBA1c is not consistently available for some of the clients; estimated average glucose level in (mg/dl) = 28.7*HbA1c-46.7 [5].

#### Event

Achieving first optimal glycemic control during the study period.

#### Survival time

The time starting from date of diagnosis with initiation of treatment to first optimal glycemic control was determined for each participant.

#### Censoring

Patients died, lost follow-up, transferred out, and completed the follow-up period without achieving optimal glycemic control.

#### Time to event

Time between initiation of treatment up to achieving first optimal glycemic control with measure of interest in month.

#### Carbohydrate counting

Practicing healthy diet at home by non-refined carbohydrate utilization and eating consistent amount of food regularly with application of food pyramid as a meal planning tool to optimize blood sugar level [29].

### Data collection tools and procedure

The data were collected from patients charted at FHCSRH and TGSTH. Data that were relevant to measure the association between times to first OGC among diabetic children were collected by two BSc nurses supervised by one senior nurse having a second degree in public health. Patient records were retrieved using their medical registration number identified in the total DM caseload in the logbook of registration follow-up form. Then medical registration number (MRN) of all diabetic pediatric patients were sorted. After that, the sample selection mechanism was a simple random sampling technique, in which each of the patients had an equal chance of being selected to be part of the study. A structured data extraction tool adapted by considering study variables such as socio-demographic, personal and clinical predictors from patients’ charts.

### Data quality assurance

Training was given to data collectors and supervisors about the objective and process of data collection by the principal investigator. Pretest was done on 5% of sample size. Then pretested data abstraction tool/check list that comprises of questions to measure the relevant variables were used to collect the necessary data from the patient medical chart by those trained data collectors. Data quality was also assured by designing proper data abstraction tools and through continuous supervision. All collected data were checked for completeness and clarity.

### Data processing and statistical analysis

The collected data was coded, entered, cleaned and stored into Epi-data version 3.1 and exported into STATA 14.0 statistical software for analysis. Descriptive statistics were presented with frequency tables, Kaplan Meier (KM) plots, and median survival times. Months are used as a time scale to calculate the time to the first OGC. The outcome of each participant was dichotomized in to censure or event (first OGC) Kaplan-Meier technique was used to measure the survival experience of diverse groups of patients by using survival curves. Log-rank tests were used to assess significant differences among survival distributions of groups for equality. After performing the Cox-proportional hazard regression, model goodness-of-fit was checked by Cox Snell residuals & assumptions were checked by using Schoenfeld residual test and graphically by using log minus log function survival curves. Bivariable analysis was performed to calculate crude hazard ratio (CHR) and to screen out potentially significant independent variables at *p*-value < 0.25 level of significance. Association between the significant independent variables and the time to first OGC were assessed using the multivariable Cox Proportional Hazard (PH) model. Adjusted hazard ratio (AHR) and 95% CI for HR were used to test significance and interpretation of results. Variables with a *p*-value < 0.05 were considered as statistically associated with the time to first OGC in months.

## Results

### Socio demographic characteristics *with censuring and event status*

Four hundred sixteen (416) medical records were reviewed; of which, 31 (7.5%) cases were excluded from the study due to pertinent data being missing. As a result, 385 clients were included in the study, which is 92.5% response rate. Median age of the study participant was 8.9 ± 1.9 years with 2.4 years mean duration of diabetes.

More than half of the patients were male (53%) and the proportion of first OGC achievement among male was (72%) which is almost proximal to female (71.3%). The majority of the patients (64.7%) were from rural areas. However, proportion of patients who achieved first OGC among rural is (68.7%) which is lower than clients from urban area residents (77.2%). Those clients having > 4 clinical visit for the last year of their follow-up had higher proportional GC (82.3%) than clients having clinical visit <=4(663%) (Table 1).

**Table 1:** Sociodemographic and institution related variable with censuring and event status among type 1 diabetic clients, Bahir Dar, Ethiopia, 2021 (*n* = 385)

Variables	Category	Event and censured status		Total
		No. of event	No.of censured	
Age group in years	<=5	83 (68%)	39 (32%)	122 (31.7%)
	> 5–10	79 (85.9%)	13 (14.1%)	92 (23.9%)
	> 10–14	114 (66.7%)	57 (33.3%)	171 (44.4%)
Sex	Male	147 (72%)	57 (27.9%)	204 (53%)
	Female	129 (71.3%)	52 (28.7%)	181 (47%)
Resident	Urban	105 ((77.2%)	31 (22.8%)	136 (35.3%)
	Rural	171 (68.7%)	78 (31.3%)	249 (64.7%)
Number of clinic visit during the last year of follow up	<=4	169 (66.3%)	86 (33.7%)	255 (66.2%)
	> 4	107 (82.3%)	23 (17.7%)	130 (33.8%)

### Incidence rate of optimal glycemic achievement rate

From 385 study participants, 276 (71.7%) of the clients achieved OGC with mean values of FBS&HA1c (112 ± 3 mg/dl, 5.6%) respectively; whereas 109 (28.3%) were censored. The lowest and the highest length of follow up were 3 and 36.4 months respectively, and the overall follow-up time in person-months was 3373.

The overall first OGC rate was 8.2 (95%CI: 7.2–9.7) per 100 person-month observation. The OGC achievement rate among male and female children with type 1 diabetes was 7.9 (95%CI: 6.7–9.3) per 100 person-month and 8.4 (95%CI: 7.1–10.0) per 100 person-month observation, respectively which is nearly comparable in both sex.

### Diabetes related variables with censuring and event status

Concerning complications, 83.4% of the patients had a history of one or more diabetes-related complications. Majority of the clients had DKA (81%) including the episodes at the time of diagnosis followed by hypoglycemia (19.7%), other complications (4.9%), and chronic complications (0.8%). The proportion of patients who achieved OGC is relatively higher among those with no history of diabetes-related complications (76.6%) as compared to those with a history of complications (70.7%). Mixed insulin (lent &regular) drugs were given to the majority of the patients (62.9%) during the initiation of treatment as compared to other regimens like Neutral Protamine Hagedom (NPH) with regular and NPH alone (20, 17.1%) respectively (Table 2).

**Table 2:** Diabetes related variable with censuring and event status among type 1 diabetic clients, Bahir Dar, Ethiopia, 2021 (*n* = 385)

Variables	Category	Event and censured status		Total
		No. of event	No. of censured	
History of diabetes related complication	NO	49 (76.6%)	15 (23.4%)	64 (16.6%)
	Yes	227 (70.7%)	94 (29.3%)	321 (83.4%)
DKA	NO	53 (72.6%)	20 (27.4%)	73 (19%)
	Yes	223 (71.5%)	89 (28.5%)	312 (81%)
Hypoglycemia	NO	211 (68.3%)	98 (31.7%)	309 (80.3%)
	Yes	65 (85.5%)	11 (14.5%)	76 (19.7%)
Chronic complication	NO	274 (71.7%)	108 (28.3%)	382 (99.2%)
	Yes	2 (66.7%)	1 (33.3%)	3 (0.8%)
Other complication^a^	NO	259 (71.9%)	101 (28%)	360 (93.5%)
	Yes	12 (63.2%)	7 (36.8%)	19 (4.9%)
More than one complication	NO	245 (72%)	95 (27.9%)	340 (88.3%)
	Yes	31 (68.9%)	14 (31.1%)	45 (11.7%)
Diabetes related hospitalization	NO	52 (74.3%)	18 (25.7%)	70 (18.2%)
	Yes	224 (71.1%)	91 (28.9%)	315 (81.8%)
Insulin Regimen	Mix(regular &lent)	154 (63.6%)	88 (36.4%)	242 (62.9%)
	NPH &regular	70 (90.9%)	7 (9%)	77 (20%)
	NPH only	52 (78.8%)	14 (21.2%)	66 (17.1%)
Non Compliance (dose omission, drug skipping, inappropriate insulin storage)	NO	219 (85.5%)	37 (14.5%)	256 (66.5%)
	Yes	56 (43.8%)	72 (56.3%)	128 (33.2%)
Duration of diabetes	< 2	75 (0.5%)	75 (0.5%)	150 (39%)
	[2–4)	80 (80.8%)	19 (19.2%)	99 (25.7%)
	> = 4	121 (89%)	15 (11%)	136 (35.3%)
Adherence to diabetic care	NO	91 (46.7%)	104 (53.3%)	195 (50.6%)
	Yes	185 (97.4%)	5 (2.6%)	190 (49.4%)
Family history of diabetes mellitus	NO	238 (71.7%)	94 (28.3%)	332 (86.2%)
	Yes	38 (71.7%)	15 (28.3%)	53 (13.8%)

^a^Other complication includes insulin injection site swelling together with lipohypertrophy and dystrophy

### Comorbidity related variables with censuring and event status

In regard to comorbidity, 69.6% of the patients had a history of comorbid illness and only 30.4% of them didn’t have a recognized history of comorbid illness. Majority of the clients had malnutrition (38.7%) followed by pneumonia (16.1%), urinary tract infection (13.8%), acute gastro enteritis (10.1%), fungal infection (7%), and upper respiratory tract infection (6.5%). Nearly half (48%) of the patients had more than one comorbid illness. The proportion of clients who achieved first OGC was higher among those with no history of comorbid illness (74.4%) than those with one or more comorbid illnesses (70.5%) (Table 3).

**Table 3:** comorbid illness related variable with censuring and event status among type 1 diabetic clients, Bahir Dar city public referral hospitals, Northwest, Ethiopia, 2021 (*n* = 385)

Variables	Category	Event and censured status		Total
		No. of event	No. of censured	
History of comorbid illness	NO	87 (74.4%)	30 (25.6%)	117 (30.4%)
	Yes	189 (70.5%)	79 (29.5%)	268 (69.6%)
Cardio vascular disease (CVD)	NO	273 (72%)	106 (28%)	379 (98.4%)
	Yes	3 (50%)	3 (50%)	6 (1.6%)
Hypertension (HTN)	NO	272 (71.8%)	107 (28.2%)	379 (98.4%)
	Yes	4 (66.7%)	2 (33.3%)	6 (1.6%)
Urinary tract infection (UTI)	NO	244 (73.5%)	88 (26.5%)	332 (86.2%)
	Yes	32 (60.4%)	21 (39.6%)	53 (13.8%)
Pneumonia (CAP)	NO	234 (72.4%)	89 (27.6%)	323 (83.9%)
	Yes	42 (67.7%)	20 (32.3%)	62 (16.1%)
Upper respiratory tract infection (URTI)	NO	264 (72.5%)	100 (27.5%)	364 (94.5%)
	Yes	15 (60%)	10 (40%)	25 (6.5%)
Acute gastro enteritis (AGE)	NO	248 (71.7%)	98 (28.3%)	346 (89.9%)
	Yes	28 (71.8%)	11 (28.2%)	39 (10.1%)
Malnutrition	NO	191 (71.5%)	76 (28.5%)	267 (69.4%)
	Yes	107 (71.8%)	42 (28.2%)	149 (38.7%)
Autoimmune disease	NO	270 (72.2%)	104 (27.8%)	374 (97.1%)
	Yes	6 (54.5%)	5 (45.5%)	11 (2.9%)
Tuberculosis (TB)	NO	273 (72%)	106 ((28%)	379 (98.4%)
	Yes	3 (50%)	3 (50%)	6 (1.6%)
Meningitis	NO	274 (73%)	101 (26.9%)	375 (97.4%)
	Yes	2 (20%)	8 (80%)	10 (2.6%)
Malaria	NO	268 (72%)	104 (28%)	372 (96.6%)
	Yes	8 (61.5%)	5 (38.5%)	13 (3.4%)
Fungal infection	NO	262 (73.2%)	96 (26.8%)	358 (93%)
	Yes	14 (51.9%)	13 (48.1%)	27 (7%)
More than one comorbid illness	NO	146 (73%)	54 (27%)	200 (51.9%)
	Yes	130 (70.3%)	55 (29.7%)	185 (48%)

### Median survival time to first optimal glycemic control

The estimated median survival time to achieve first OGC was 8 months with an inter-quartile range of (6.9–8.9). The median survival time to first OGC among type one diabetic children varied by various categories of predictors. For example, the median survival time to achieve first OGC among under 5 children was 6.8, where as in above 5–10, and > 10–14 years was 8, 8.5, respectively (Table 4).

**Table 4:** comparisons of optimal glycemic control among type 1 DM clients, Bahir Dar city public referral hospitals, Northwest, Ethiopia, 2021 (*n* = 385)

Variables	Category	Test of equality over groups		Log rank		
		Median survival time (months)	Mean survival time (months)			
				X^2^	DF	*P*-value
Age group in years	<=5	6.8	8.5	6.05	2	0.0486
	> 5–10	8	9.8			
	> 10–14	8.5	10.2			
Sex	Male	8.5	9.9	0.92	1	0.3378
	Female	7.2	9.2			
Resident	Urban	7.6	9.6	0.02	1	0.8911
	Rural	8	9.6			
Education status of children	KG/not started	7.1	8.9	11.23	2	0.0036
	Primary school	9	10.6			
	High school	14.8	13			
Family history of diabetes	NO	7.8	8.7	0.28	1	0.5987
	Yes	8	9.4			
Number of clinic visit	<=4	7.7	8.5	1.31	1	0.2521
	> 4	8	9.4			
Adherence to diabetic care	NO	14.9	10.9	131.75	1	< 0.0001
	Yes	5.7	6.7			
Insulin regimen	Mixed (lent &Regular)	7.1	8.4	15.87	2	0.0004
	NPH& Regular	9.2	10.1			
	NPH only	9.8	12.3			
Duration of Diabetes in year	< 2	5.5	6.2	54.93	2	< 0.0001
	[2–4)	8.6	10			
	> = 4	11.1	11.4			
Carbohydrate count	NO	10.2	11.1	40.26	1	< 0.0001
	Yes	5.5	6.9			
Noncompliance	NO	6.4	8.2	42.30	1	< 0.0001
	Yes	14.8	14.9			
Diabetes related acute complication	NO	7.7	9.5	2.94	1	0.0862
	Yes	8	9.6			
Diabetic ketoacidosis	NO	6.2	9.5	0.12	1	0.7289
	Yes	8	9.6			
Chronic complication	NO	7.8	8.7	0.59	1	0.4434
	Yes	12.1	18.5			
Other complication	NO	7.8	9.5	1.02	1	0.3131
	Yes	10.2	11.3			
More than one complication	NO	7.8	9.3	0.21	1	0.6448
	Yes	8.9	10			
History of comorbidity	NO	6.3	8.3	10.85	1	0.0010
	Yes	8.9	10.1			
Wasting	NO	8.2	8.9	1.07	1	0.3003
	Yes	6.8	8.6			
Stunting	NO	7.8	8.8	0.15	1	0.7019
	Yes	9.8	8.4			
Cardio vascular disease	NO	7.8	8.8	0.01	1	0.9229
	Yes	12.1	10.2			
Pneumonia	NO	7.7	8.9	0.89		0.3460
	Yes	9	8.1			
Acute gastro enteritis	NO	7.7	9.4	2.05	1	0.1524
	Yes	10.2	11.5			
More than one comorbid illness	NO	7.7	9.5	0.21	1	0.6448
	Yes	8.7	9.7			

*X*^*2*^ chi-square, *DF* Degree of freedom, *KG* kindergarten

### Survival estimates for time to first optimal glycemic control

**Fig. 1:**
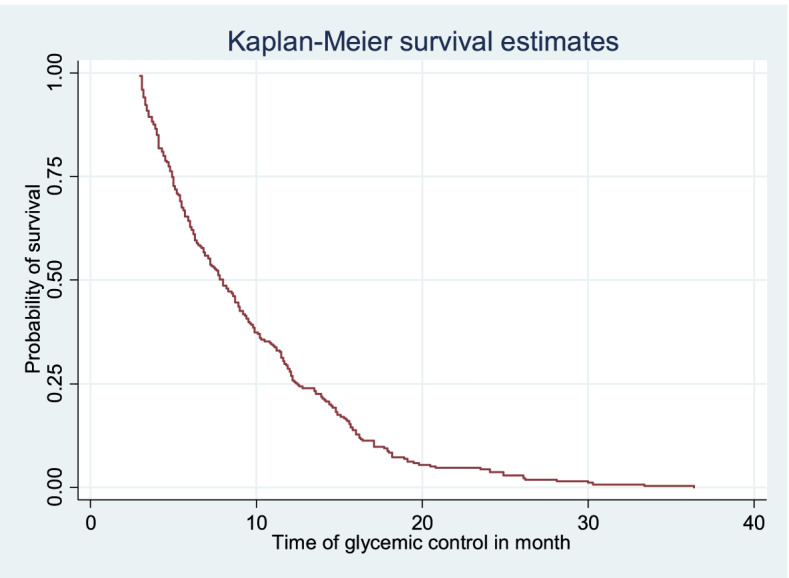
Kaplan-Meier survival estimate of time to first optimal glycemic control among type 1 diabetic children having follow up at Bahir Dar city public referral hospitals, 2021

**Fig. 2:**
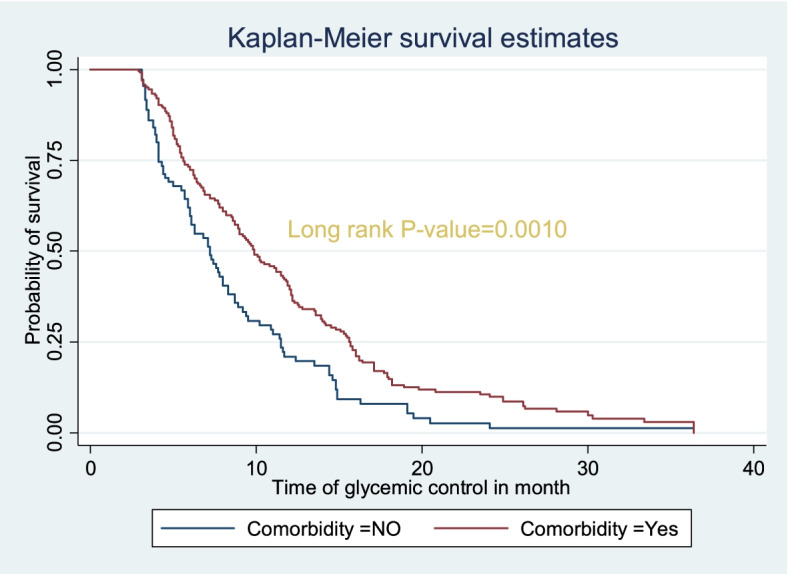
Kaplan Meier survival estimate for time to optimal glycemic control among type 1 diabetic children with history of comorbidity in Bahir Dar city public referral hospitals, Northwest, Ethiopia, 2021

The survival status of children with type 1 diabetes was estimated by the Kaplan-Meier survival curve. The curve tends to decrease rapidly within the first 1 year, indicating that most children achieved their first OGC within this time (Fig. 1). The survival estimates of clients varied in relation to different predictors (Fig. 2).

### Comparison of survival experience

The long rank test was used to assess differences in equality of survival distribution among diverse groups. The median survival time to achieve first OGC among clients in the age groups of <=5 years showed shorter median time to achieve first OGC (6.8 months) as compared with patients whose age group between 6 and 10 years (8 months) and 11–14 years (8.5 months). And the survival time was significantly different among the age groups (X^2^(2)) = 6.05; *P*-value = 0.0486) whereas, the median survival time to achieve first OGC among male participants showed a relatively longer time (8.5 months) than females (7.2 months). But the long rank test was not statistically significant (X^2^(1)) = 0.92, *p*-value = 0.3378) (Table 4).

**Fig. 3:**
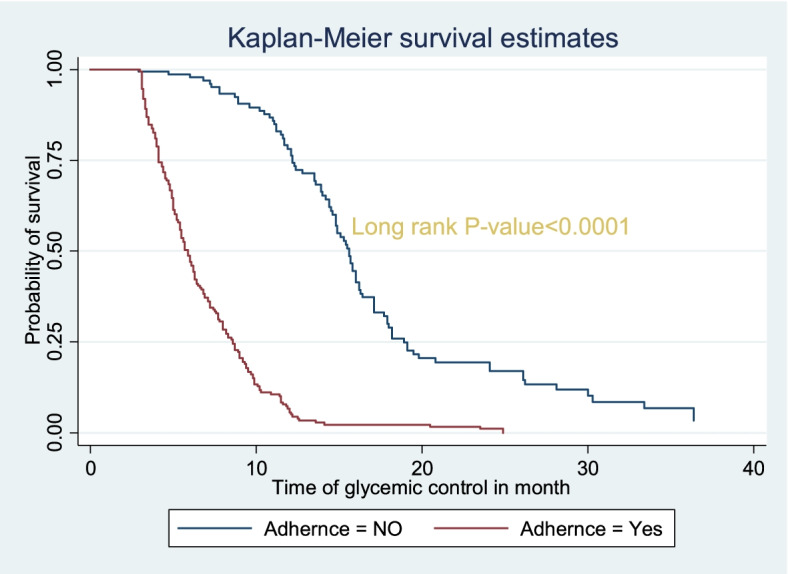
survival and hazard function of adherence by time (in month), Bahir Dar city public referral hospitals, Northwest, Ethiopia, 2021

Regarding adherence, those clients who adhered to the management had a shorter duration of time (5.7 months) to achieve first OGC than those who didn’t adhere towards the management of the disease (14.9 months). The long rank test was statistically significant (X^2^(1)) = 131.75, *P*-value < 0.0001). The Kaplan Meier survival function showed that, clients with adherence have a satisfactory survival experience by achieving their glycemic targets early in time. The figure also showed that, clients direct chance of achieving first OGC increases for both groups as the duration of treatment increases (Fig. 3).

Those patients having a comorbid illness appear to extend the time to first OGC. The median survival time to achieve OGC was shorter among patients with no history of comorbid illness (6.3 months) than patients who had comorbid illness (8.9 months) with statistically significant differences among the groups (X^2^(1)) = 10.85, *P*-value = 0.0010) (Table 4).

However, no statistically significant differences were shown for sex, residence, family history of diabetes mellitus, number of clinic visits, DKA as presentation, and being malnourished in determining time to first OGC (Table 4).

### Results of multivariable cox proportional hazard model

**Fig. 4:**
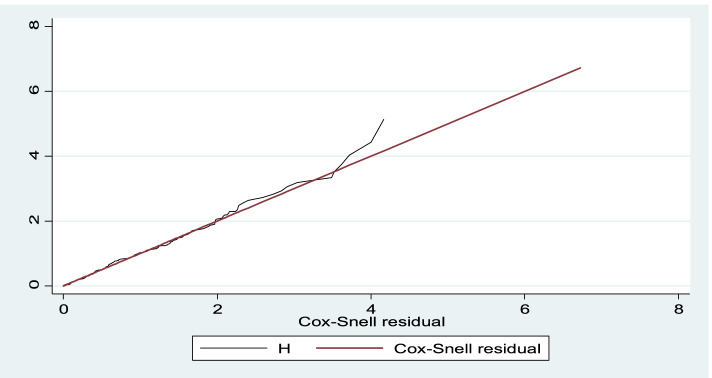
Model goodness of fit by cox Snell residual among type 1 DM clients, Bahir Dar city public referral hospitals, Northwest, Ethiopia, 2021 (*n* = 385)

Goodness of fit checked by cox Snell residual by plotting cox Snell residual against the cumulative hazard function. As a residuals follow unit of exponential distribution or a linear line through the origin with a unit gradient, which indicates a well-fitted model to the observed data point and expected value (Fig. 4).

**Fig. 5:**
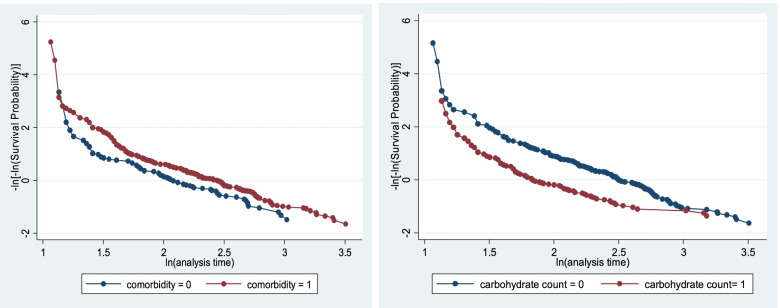
log of minus the Log of survival function by comorbidity and carbohydrate count for time to first optimal glycemic control among type 1 diabetic children, Bahir Dar, 2021

The proportional assumption of cox proportional hazard model was tested by using Schoen field residual test/global test (0.5368) and graphically by using log minus log function on Stata version 14.2 (Fig. 5). The survival curve looks parallel throughout the study time; which shows equitable fitting to the proportional hazard assumption (Fig. 5).

The independent variables such as age, client educational status, primary care giver, dose of insulin at initiation of treatment, duration of diabetes, first insulin regimen, current insulin regimen, frequency of GC, carbohydrate count, exercise, noncompliance, adherence, diabetes related acute complication, having history of comorbidity were significantly associated with time to first OGC at the point less than 0.25 level of significance from bivariable analysis. However, age > 10–14 years, duration of DM ≥4 years, dose of insulin at initiation of treatment, overweight, having primary care giver, adherence to DM care, carbohydrate counting and history of comorbidity were found to be significantly associated with time to first OGC in the multivariable cox regression hazard model less than 5% level of significance.

The presence of interaction among independent variables was checked by multicollinearity test but there was no significant interaction as it was confirmed by the value of each variables variance inflation factor (VIF) which is less than seven (mean value of VIF = 2.52).

Consequently, after adjusting other predictor, the hazard of achieving OGC among the age groups > 10–14 years were lower by 67.6% as compared with the age groups of the client ≤5 years (AHR = 0.324;95%CI = 0.192–0.546); The rate of OGC decreases by 3.6% as weight increases by one unit (AHR = 0.964;95%CI = 0.939–0.989); The hazard of achieving OGC among clients living with diabetes equal to or greater than 4 years were lower by 35.8% as compared with clients living with diabetes for less than 2 years (AHR = 0.642;95%CI = 0.436–0.944); Likewise, the hazard of achieving OGC among clients with a history of comorbid illness was lower by 27.8% compared to clients with no history of comorbid illness (AHR = 0.722; 95%CI = 0.530–0.981); This means, the time needed to reach OGC among clients with a history of comorbid illness was significantly longer compared with clients with no history of comorbid illness.

However, the rate of achieving first OGC among clients who adhere to diabetic care had a 9.7 times greater increment than clients who didn’t adhered to diabetic management (AHR = 9.723;95%CI = 6.094–15.513); Dose of insulin at initiation of treatment increases first OGC achievement rate by 1.053 times as dose of insulin increases by one unit (AHR = 1.053;95%CI = 1.029–1.078); The rate of achieving first OGC among clients who cared by both mother and father were 2.092 times greater than the clients supported by their mother alone (AHR = 2.092;95%CI = 1.397–3.132); Similarly, the rate of achieving first OGC among clients who used carbohydrate count had a 2.433 times greater increment than clients who didn’t use it (AHR = 2.433;95%CI = 1.124–5.263) (Table 5).

**Table 5:** Results for the final cox regression hazard model among type 1DM clients Bahir Dar city public referral hospitals, Northwest, Ethiopia, 2021 (*n* = 385)

Variable	CHR (95%CI)	AHR (95% CI)	*P*-value
Insulin dose at initiation of Rx	0.982 (0.969–0.993)^a^	1.053 (1.029–1.078)	**< 0.001**^b^
Weight of the client	0.978 (0.965–0.992)^a^	0.964 (0.939–0.989)	**0.005**^b^
Age group in years at diagnosis			
<=5®			
> 5–10	0.802 (0.587–1.097)	0.926 (0.619–1.384)	0.707
> 10–14	0.599 (0.448–0.801)^a^	0.324 (0.192–0.546)	**< 0.001**^b^
Sex of the participant			
Male®			
Female	1.116 (0.879–1.416)		
Resident			
Urban®			
Rural	1.010 (0.790–1.292)		
Primary care giver			
Mother alone®			
Mother and Father	0.848 (0.617–1.165)	2.092 (1.397–3.132)	**< 0.001**^b^
Father alone	0.824 (0.493–1.378)	1.171 (0.631–2.171)	0.617
Other	0.685 (0.475–0.988)^a^	0.801 (0.491–1.305)	0.372
Educational status of children			
KG/not started®			
Primary school	0.746 (0.527–1.057)	0.868 (0.574–1.314)	0.505
High school	0.684 (0.471–0.992)^a^	1.333(0.745–2.386)	0.333
Insulin regimen			
Lent& regular®			
NPH& regular	0.840 (0.631–1.118)^a^	0.757 (.538–1.066)	0.111
NPH alone	0.704 (0.511–0.970)^a^	1.305 (0.856–1.990)	0.216
Carbohydrate counting			
NO®			
Yes	4.173(2.332–7.468)^a^	2.433(1.124–5.263)	**0.024**^b^
Frequency of glycemic control per day			
< 3®			
> = 3	1.904 (1.409–2.574)^a^	1.259 (0.887–1.788)	0.198
Physical exercise			
NO®			
Yes	2.574 (1.991–3.326)^a^	1.178 (0.841–1.649)	0.341
Noncompliance behavior assessed by clinician at health care visit			
NO®			
Yes	0.334 (0.248–0.451)^a^	1.222 (.805–1.853)	0.346
Adherence to diabetic care			
NO®			
Yes	6.522 (4.901–8.679)^a^	9.723(6.094–15.513)	**< 0.001**^b^
Duration of DM in years			
< 2®			
[2–4)	0.559 (0.401–0.781)	0.736 (0.509–1.063)	0.102
> = 4	0.486 (0.356–0.664)^a^	0.642 (0.436–0.944)	**0.024**^b^
Diabetes related acute complication			
NO®			
Yes	1.591 (1.031–2.457)^a^	1.084 (.653–1.799)	0.755
Other complication			
NO®			
Yes	0.746 (0.456–1.221)		
History of comorbidity			
NO®			
Yes	0.627 (0.484–0.811)^a^	0.722 (0.530–0.981)	**0.038**^b^

*CHR* Crude hazard ratio, *AHR* Adjusted hazard ratio, *Rx* Treatment,**®** Reference group and ^a^ & ^b^ indicates statistically significant variable with bivariable & multivariable cox regression hazard model respectively

## Discussion

The purpose of this study was to estimate time to first OGC and identify its predicting factors among type one diabetic children less than 15 years in Bahir Dar city public referral hospitals. In this study, the median survival time to achieve first OGC was 8 months with the overall incidence rate of optimal glycemic control 8.2 (95%CI: 7.2–9.2) per 100 person-month observation.

Although no clear cut-off point is set about when to reach first OGC, most of clinical experts expect the first diagnosed type one diabetic clients to achieve glycemic targets within 3 months of diagnosis, following to the trends to have frequent visit and strict follow-up with proper diabetic care [6]. Based on the follow-up measurement tool that the hospital uses, the findings of this study point out long time to achieve first glycemic targets among type 1 diabetic children.

In the meantime, there is no former similar study; as a result, the finding of this study is compared with studies conducted with exactly analogous factors that affect OGC among type 1 diabetic children from diverse literature in different parts of the country.

In regard to predictors, the age of the participant was found to be significantly associated with variables that determined the time to first OGC. The study showed that, the time needed to reach first OGC is longer among clients of age group > 10–14 years compared to clients in the age group≤5 years (AHR = 0.324,95%CI = 0.192–0.546), indicating that for children older than 10 years, the rate of achieving OGC decreases as age increases which is in line with study done in Tanzania,Bulgaria, Iraq, Taiwan and Jordan [12, 16, 32–34]. This can be due to the fact that as a child develops, he/she under-goes a varieties of physical and life style changes [24]. In addition to this, it can be also be due to hormonal effect at the pubertal age of the child and decline in parental supervision over different clinical aspects of diabetes care in the adolescents [16, 32].

Weight of the client was also significantly associated with time to first OGC. Rate of glycemic achievement decreases by 3.6% as weight increases by one unit, which means the weight of the client is 0.964 times less likely associated with OGC achievement rate. This could be due to, weight gain may contribute to increased insulin resistance and cardio-metabolic risk such as increased dyslipidemia and blood pressure [35]. It is in line with another controlled study among T1DM patients which stated previously as “normal weight preschool children have better GC than age-matched overweight children” [36, 37]. It can significantly suggest that, body weight status may impede achievement of glycemic targets within the expected time in this group of patients. Therefore, having regular exercise which is non-strenuous can be encouraged. The recommendation is supported by the study conducted in United Kingdom and the authors of International Society of Pediatrics and Adolescents Diabetes (ISPAD) guidelines revised since 2018 [3, 28].

Dose of insulin at initiation of treatment increases first OGC achievement rate by 1.053 times as dose of insulin increases by one unit. This finding is supported by the study done in many countries such as India, China, Germany, Austria, and Luxembourg [38–41]. Optimal glycemic control with insulin therapy for T1DM is fundamental which should be aiming to achieve good glycemic control with achievement of HbA1c < 7.5% with in short period of time [38]. The use of either Pediatrics continuous glucose monitoring or increasing the frequency of insulin injection can increased the chance of lowering HbA1c/average FBG level and improves time in target regardless of insulin delivery modality [39].Implementing strict insulin regimens could improve glycemic control in people with T1DM; In other ways, numerous daily subcutaneous insulin injections route using syringe and vial and sometimes insulin pens remains the most predictable route for insulin administration among diabetic children [41]. The find also agrees with the study reported in Kansas City, United States, highlighting that, early initiation of either an insulin pump or CGM in children diagnosed with T1D can help to improve child HbA1c levels within the first 12 months of diabetes [30].

This study also showed that, having a primary care giver during the follow-up period was significantly associated with OGC. Especially those clients whose caregiver mother and father were two times more likely to be associated with first OGC as compared with clients supported by their mothers alone. The finding was supported by the study conducted in Tanzania and middle east Jordan [13, 16]. In the presence of ongoing family support, supervision and Proactive efforts to prevent deterioration can enhance adaptation to type 1 diabetes management [13]. Sustained parental involvement in the management of diabetes care is important as children transition into adolescence, and the best outcomes are evident in this study when this involvement occurs kindly in collaborative manner (involving both mother and father the care process). The finding also agrees with the study done in Netherlands which underlined that, management of type 1 diabetes in children by encouraging more parental responsibility were significantly associated with better glycemic control over time [18, 42].

In regard to adherence to diabetic care, those clients with adherence had 9.7 fold of instantaneous chance of increasing their glycemic achievement rate as compared with those clients with no adherence to their diabetes management. Which is in line with the study conducted in Ethiopia entitled with incidence of DKA and its predictors among type one diabetic children [27]. The finding implies that, adherence to the diabetes treatment regimen can possibly the most common reason for good health outcomes/optimal glycemic control with in short period of time. Correspondingly, those clients well adhered to Diet counseling/practicing proper carbohydrate counting specifically on food pyramid and non-refined carbohydrate utilization were found to have increasing their glycemic achievement rate by 2.4 folds as compared with those clients with no habit of practicing healthy diet at home and the finding is in line with the study conducted in Uganda, Australia and brazil [21, 29, 37, 43]. In supporting this finding, HbA1c was significantly higher in children offered food in a grazing pattern compared with those offered regular meals [29]. Which implies that, Individuals on insulin therapy need to eat at consistent times corresponding with the time-actions of insulin, with regular monitoring of blood glucose levels, and insulin doses with equivalent to the food eaten [43]. And therefore, carbohydrate counting is important in preventing diabetes, managing existing diabetes, and preventing, or at least slowing, the rate of development of diabetes complications.

Duration of diabetes was also significantly associated with time to first OGC in this study. Those clients living with diabetes for more than or equal to 4 years were 35.8% less likely to achieve OGC as compared with clients who were living with diabetes for less than 2 years. This could be due to age maturation with advancement of the disease following diabetic duration as it was explained above [16, 24, 32]. This finding is similar with the study done in Tanzania [27] but different with the study done in Cameron [44].

In addition to the above factors, having a comorbid illness is another important predictor that can affect time to OGC. The rate of achieving OGC among clients with a history of comorbid illness was 27.8% times less likely as compared with clients with no comorbid illness. This is because having a comorbid illness has an influence on diabetes disease progress with impairment of glucose metabolism possibly leading to deterioration of GC. Comorbid illness such as infection might also cause high level of counteracting hormones which triggering an episode of hyperglycemia and could also be due to the effect of taking many drugs which can lead to drug interaction and also can decrease drug adherence which interferes with drug effectiveness. This finding is in line with studies conducted in Saudi Arabia, Brazil, and University of California, San Francisco [17, 18, 21, 22, 45, 46].

In general, this study can bring out positive implications for clinical care, health service management and researches with in an area of diabetic specialization. Clinically, the health care worker can identify predictors associated with time to first optimal glycemic control among type one diabetic children at clinical setup. Health care managers can access current evidences about time when the patient reach on optimal glycemic targets and to take remedial action to strengthen service delivery by the clinicians. Researcher can also motivated to conduct further researches in this area by taking this study as preliminary findings.

## Conclusion and recommendation

The median survival time to first OGC in this study was longer than expected, which might imply that clients are being exposed to more risk of both acute and chronic complications of type one diabetes mellitus. This increased risk remains higher for those clients achieving their glycemic control with in long period of time compared to those who achieved optimal glycemic control in a short period of time Age > 10–14 years, overweight, having primary caregiver, insulin dose, duration of diabetes, adherence to care, and carbohydrate counting including history of comorbidity were determinant factors. Therefore, clinicians should give attention for weight reduction, comorbid illness, dose of insulin during initiation of treatment, parent counseling about adherence to overall diabetic care focusing on insulin drugs and how to audit their children’s diet as prescription helps to reduce the length of glycemic control.

### Strength and limitation of the study

#### Strength of the study

One of the strengths of this study is that, it provides current evidence on predictors associated with time to first optimal glycemic control among type 1 diabetic children, which, to my knowledge, have not previously been documented in Ethiopia.

#### Limitation of the study

Since the data were collected from medical records, variables like parental socio-economic factors cannot be addressed through card review, which may affect the outcome of the study. FBS measurements obtained from medical records might be subjected to measurement errors that lead to an underestimated or overestimated of the result. However, effort was made to overcome these issues by taking the mean values of 3 month consecutive value of FBS measurements.

## Data Availability

Data will be available upon request from the corresponding author.

## Abbreviations

DCCT: Diabetes Control and Complications Trial
DKA: Diabetic ketoacidosis
DM: Diabetes Mellitus
EDIC: Epidemiology of Diabetes Interventions and Complications
FBS: Fasting Blood Glucose
GC: Glycemic Control
HbA1c: Glycated Hemoglobin A1C
IDF: International Diabetic Federation
ISPAD: International Society of Pediatrics and Adolescent Diabetes
MRN: Medical Registration Number
OGC: Optimal Glycemic Control
T1DM: Type 1 Diabetes Mellitus
WHO: World Health Organization

## References

[1] OMS. Global report on diabetes. Isbn 2016;978:6–86. Available from: http://www.who.int/about/licensing/copyright_form/index.html%0Ahttp://www.who.int/about/licensing/copyright_form/index.html%0Ahttps://apps.who.int/iris/handle/10665/204871%0A; http://www.who.int/about/licensing/. Accessed 17 Dec 2020.

[2] Leulseged TW, Ayele BT (2019). Time to optimal glycaemic control and prognostic factors among type 2 diabetes mellitus patients in public teaching hospitals in Addis Ababa, Ethiopia. PLoS One..

[3] DiMeglio LA, Acerini CL, Codner E, Craig ME, Hofer SE, Pillay K, et al. ISPAD Clinical Practice Consensus Guidelines 2018: Glycemic control targets and glucose monitoring for children, adolescents, and young adults with diabetes. Pediatr Diabetes. 2018;19 (July):105–14. https://onlinelibrary.wiley.com/doi/full/10.1111/pedi.12737/. Accessed 17 Dec 2020. 10.1111/pedi.12737 30058221

[4] Shah AS, Nadeau KJ (2020). The changing face of paediatric diabetes. Diabetologia..

[5] Bozkaya G, Ozgu E, Karaca B (2010). The association between estimated average glucose levels and fasting plasma glucose levels. Clinics..

[6] L. N, B. A, W. M, K. P. Determinants of outcome of children with type 1 diabetes in Cameroon. Horm Res Paediatr 2015;84:185. Available from: http://www.embase.com/search/results?subaction=viewrecord&from=export&id=L72085656%0A; 10.1159/000437032

[7] Ramírez-Mendoza F, González JE, Gasca E, Camacho M, Cruz MV, Caraveo D (2020). Time in range and HbA1C after 6 months with a multidisciplinary program for children and adolescents with diabetes mellitus, real world data from Mexico City. Pediatr Diabetes.

[8] Shibeshi MS, Fantahun B, Kebede T, Tilahun B (2016). Pediatric diabetic retinopathy: experience of a tertiary hospital in Ethiopia. BMC Res Notes..

[9] Niba LL, Aulinger B, Mbacham WF, Parhofer KG (2017). Predictors of glucose control in children and adolescents with type 1 diabetes: results of a cross-sectional study in Cameroon. BMC Res Notes..

[10] Yazidi M, Chihaoui M, Chaker F, Rjeb O, Slimane H (2016). Factors predicting glycemic control in type 1 diabetic patient. Open Med J.

[11] Abebe SM, Berhane Y, Worku A, Alemu S, Mesfin N (2015). Level of sustained glycemic control and associated factors among patients with diabetes mellitus in Ethiopia: A hospital-based cross-sectional study. Diabetes Metab Syndr Obes Targets Ther.

[12] Mclarty RP, Alloyce JP, Chitema GG, Msuya LJ. Glycemic control , associated factors , acute complications of Type 1 Diabetes Mellitus in children , adolescents and young adults in Tanzania. 2020;(July):1–8. 10.1002/edm2.200 PMC8029575 33855206

[13] Noorani M, Ramaiya K, Manji K (2016). Glycaemic control in type 1 diabetes mellitus among children and adolescents in a resource limited setting in Dar Es Salaam- Tanzania. BMC Endocr Disord.

[14] Ngwiri T, Were F, Predieri B, Ngugi P, Iughetti L. Glycemic control in Kenyan children and adolescents with type 1 diabetes mellitus. Int J Endocrinol. 2015. 10.1155/2015/761759. 10.1155/2015/761759 PMC4606130 26494998

[15] Taha Z, Eltoum Z, Washi S (2018). Predictors of glucose control in children and adolescents with type 1 diabetes: results of a cross-sectional study in Khartoum, Sudan. Open Access Maced J Med Sci.

[16] Alassaf A, Odeh R, Gharaibeh L, Ibrahim S, Ajlouni K (2019). Personal and clinical predictors of poor metabolic control in children with type 1 diabetes in Jordan. J Diabetes Res.

[17] Al-Agha AE, Alafif M, Abd-Elhameed IA (2015). Glycemic control, complications, and associated autoimmune diseases in children and adolescents with type 1 diabetes in Jeddah, Saudi Arabia. Saudi Med J.

[18] K. K. Hood and D. Ph, “Predictors of deteriorations in diabetes management and control in adolescents with type 1 diabetes. 2015;52(1): 28–34**.** https://cdr.lib.unc.edu/downloads/v405sh74p/. Accessed 17 Dec 2020. 10.1016/j.jadohealth.2012.05.009 PMC4467546 23260831

[19] Archinkova M, Konstantinova M, Savova R, Iotova V, Petrova C, Kaleva N, et al. Glycemic control in type 1 diabetes mellitus among Bulgarian children and adolescents: the results from the first and the second national examination of HbA1c. Biotechnol Biotechnol Equip. 2017;31(6):1198–203. Available from: 10.1080/13102818.2017.1379360.

[20] Gebreyohannes EA, Netere AK, Belachew SA (2019). Glycemic control among diabetic patients in Ethiopia: A systematic review and meta-analysis. PLoS One.

[21] Andrade CS, Ribeiro GS, Santos CAST, Neves RCS, Moreira ED (2017). Factors associated with high levels of glycated haemoglobin in patients with type 1 diabetes: A multicentre study in Brazil. BMJ Open.

[22] Krzewska A, Ben-Skowronek I. Effect of associated autoimmune diseases on type 1 diabetes mellitus incidence and metabolic control in children and adolescents. Biomed Res Int. 2016;2016:12. 10.1155/2016/6219730. 10.1155/2016/6219730 PMC4971288 27525273

[23] Setoodeh A, Mostafavi F, Rabbani A, Hedayat T. Female Sex as a Risk Factor for Glycemic Control and Complications in Iranian Patients with Type One Diabetes Mellitus 2011;21(3):373–378. PMC3446184 23056816

[24] Smokovski I. Managing diabetes in low income countries. Manag Diabetes Low Income Ctries. 2021.

[25] Msanga D, Reis K, Kayange N, Bakalemwa R, Kidenya B, Hau D (2020). Diabetic microvascular complications among children and adolescents in northwestern Tanzania: A cross-sectional study. Ann Glob Heal.

[26] Shiferaw WS, Akalu TY, Aynalem YA. Chronic kidney disease among diabetes patients in Ethiopia: a systematic review and Meta-analysis. Int J Nephrol. 2020;2020:15. 10.1155/2020/8890331. 10.1155/2020/8890331 PMC7569456 33101733

[27] Zeleke H, Murugan R, Wondwossen K. Incidence and predictors of diabetic ketoacidosis among children with diabetes in west and east gojjam zone referral hospitals north west Ethiopia. 2019; 10.1186/s13052-020-00930-4 PMC7640382 33143741

[28] Taylor GS, Smith K, Capper TE, Scragg JH, Bashir A, Flatt A (2020). Postexercise glycemic control in type 1 diabetes is associated with residual b-cell function. Diabetes Care.

[29] Seckold R, Howley P, King BR, et al. Dietary intake and eating patterns of young children with type 1 diabetes achieving glycemic targets. BMJ Open Diab Res Care. 2019;7:e000663. 10.1136/bmjdrc-2019-000663. 10.1136/bmjdrc-2019-000663 PMC6606069 31321060

[30] Patton SR, Noser AE, Youngkin EM, Majidi S, Clements MA, Al PET (2019). Early initiation of diabetes devices relates to improved glycemic control in children with recent-onset type 1. Diabetes Mellitus.

[31] MOH. Guidelines on Clinical and Programmatic Management of Major Non Communicable Diseases. 2016;220. https://extranet.who.int/ncdccs/Data/ETH_D1_National%20NCD%20Guideline/. Accessed 17 Dec 2020.

[32] Rewers MJ, Pillay K, de Beaufort C, Craig ME, Hanas R, Acerini CL, et al. Assessment and monitoring of glycemic control in children and adolescents with diabetes. Pediatr Diabetes 2014;15(SUPPL.20):102–14. 10.1111/pedi.12190 25182311

[33] Governorate S. Original article factors affecting glycemic control in type 1 Diabetes Mellitus among. 2019;8(2).

[34] Hsiao YT, Cheng WC, Liao WC, Lin CL, Shen TC, Chen WC (2015). Type 1 diabetes and increased risk of subsequent asthma: A nationwide population-based cohort study. Med (United States).

[35] Jaja TC, Yarhere IE (2019). Dyslipidaemia in Nigerian children and adolescents with diabetes mellitus: prevalence and associated risk factors. Int J Diabetes Metab.

[36] Nansel TR, Lipsky LM, Iannotti RJ (2014). Cross-sectional and longitudinal relationships of body mass index with glycemic control in children and adolescents with type 1 diabetes mellitus. Multicenter Study.

[37] Kerkeni L, Ruano P, Delgado LL, Picco S, Villegas L, Tonelli F, et al. Nutritional Management in Type 1 Diabetes Mellitus. Intech. 2016;(tourism):13. Available from: https://www.intechopen.com/books/advanced-biometric-technologies/liveness-detection-in-biometrics.

[38] Wangnoo SK (2015). Initiating insulin therapy in children and adolescents with type 1 diabetes mellitus. Indian J Endocrinol Metab.

[39] Maiorino MI, Signoriello S, Maio A, Chiodini P, Bellastella G, Scappaticcio L (2020). Effects of continuous glucose monitoringonmetricsofglycemic control in diabetes: A systematic review with Meta-analysis of randomized controlled trials. Diabetes Care.

[40] Klatman EL, Ogle GD (2020). Access to insulin delivery devices and glycated haemoglobin in lower-income countries. World J Diabetes.

[41] Lin K, Yang X, Yin G, Lin S. Diabetes self-care activities and health-related quality-of-life of individuals with type 1 diabetes mellitus in Shantou, China. 2016; 10.1177/0300060515597933 PMC5536571 26658458

[42] Rotteveel J, Waarde WMB, Houdijk ECAM, Nuboer R, Winterdijk P, Snoek FJ. Youth With Type 1 Diabetes Taking Responsibility for Self- Management: The Importance of Executive Functioning in Achieving Glycemic Control Results From the Longitudinal DINO Study 2019;42(February):225–231. 10.2337/dc18-1143 30552132

[43] Ndahura NB, Munga J, Kimiywe J, Mupere E. Caregivers ’ Nutrition Knowledge and Dietary Intake of Type 1 Diabetic Children Aged 3–14 Years in Uganda. 2021;127–37. 10.2147/DMSO.S285979 PMC7813451 33469330

[44] Djonou C, Tankeu AT, Dehayem MY, Tcheutchoua DN, Mbanya JC, Sobngwi E. Glycemic control and correlates in a group of sub Saharan type 1 diabetes adolescents 11 medical and health sciences 1117 public health and health services. BMC Res Notes. 2019;12(1):1–5. Available from: 10.1186/s13104-019-4054-1. 10.1186/s13104-019-4054-1 PMC6341641 30670077

[45] Calliari LE, Almeida FJ, Noronha RM (2020). Infections in children with diabetes. J Pediatr.

[46] Chiang JL, Maahs DM, Garvey KC, Hood KK, Laffel LM, Weinzimer SA, et al. Type 1 diabetes in children and Adolescents: A position statement by the American Diabetes Association 2018;41(September):2026–44. https://care.diabetesjournals.org/content/41/9/2026.article-info/. Accessed 17 Dec 2020. 10.2337/dci18-0023 PMC6105320 30093549

